# Gelatin-sodium alginate hydrogel infused with *Onosma dichroanthum* Boiss. root extract: Preparation, characterization, and application in wound dressing

**DOI:** 10.1016/j.bbrep.2025.102236

**Published:** 2025-09-09

**Authors:** Hadi Hossainpour, Soheila Zare, Hosna Alvandi, Ramin Abiri, Faranak Aghaz, Elham Arkan, Amirhooshang Alvandi

**Affiliations:** a Department of Microbiology, School of Medicine, Ardabil University of Medical Sciences, Ardabil, Iran; b Zoonoses Research Center, Ardabil University of Medical Sciences, Ardabil, Iran; c Nano Drug Delivery Research Center, Health Technology Institute, Kermanshah University of Medical Sciences, Kermanshah, Iran; d Nanobiotechnology Department, Faculty of Innovative Science and Technology, Razi University, Kermanshah, Iran; e Fertility and Infertility Research Center, Research Institute for Health Technology, Kermanshah University of Medical Sciences, Kermanshah, Iran; f Medical Technology Research Center, Health Technology Institute, Kermanshah University of Medical Sciences, Kermanshah, Iran

**Keywords:** Alginate, Gelatin, Hydrogels, Wound healing, *Onosma dichroanthum boiss*

## Abstract

**Background:**

Chronic and acute wounds remain a major clinical challenge, often complicated by infection, delayed healing, and patient morbidity. Hydrogels have emerged as promising wound dressings due to their moist environment maintenance, exudate management, and capacity to deliver bioactive agents. However, many hydrogel systems rely on synthetic components or show limited antimicrobial efficacy and mechanical adaptability to diverse wound types. Natural polymers such as alginate and gelatin offer biocompatibility and tunable properties but frequently require enhancements to achieve optimal elasticity, degradation rates, and biological function. There is a critical need for a biocompatible, mechanically robust, and antimicrobial hydrogel platform that can be easily loaded with plant-derived bioactives to promote rapid and safe wound closure. The focus of this study was to prepare and characterize GEL/SA hydrogels incorporated with *O. dichroanthum* root extract for wound dressing applications.

**Materials and methods:**

The hydrogel was synthesized using the casting and cross-linking method. Its physicochemical and antimicrobial properties were thoroughly evaluated.

**Results:**

The GEL/SA–*O. dichroanthum* hydrogel demonstrated strong elasticity and promoted effective biodegradation, while GEL/SA–*O. dichroanthum* hydrogels exhibited excellent hemocompatibility. Additionally, these hydrogels showed significant antibacterial activity against *Staphylococcus aureus* (9 mm) and potent antifungal effects against *Candida albicans* (10 mm).

**Conclusion:**

This study presents a GEL/SA hydrogel incorporated with *Onosma dichroanthum* root extract, achieving a unique combination of features: A dual-polymer natural matrix (gelatin-alginate) with tuned cross-linking that yields high mechanical stability while exhibiting controlled relaxation and enhanced elasticity after extract incorporation. Integration of *O. dichroanthum* root extract as a bioactive wound-healing constituent, delivering both anti-inflammatory and antimicrobial effects directly at the wound site. Demonstration of improved biodegradation kinetics and excellent hemocompatibility without compromising structural integrity or biocompatibility. Demonstration of significant antibacterial activity against *Staphylococcus aureus* and potent antifungal effects against *Candida albicans*, addressing common wound-infection challenges. A scalable, casting-and-crosslinking fabrication approach compatible with clinical translation and potential for combination with additional therapeutic agents.

## Highlights

• GEL/SA-*O. dichroanthum* hydrogels exhibited excellent hemocompatibility.
• GEL/SA-*O. dichroanthum* hydrogels showed significant antibacterial activity against *Staphylococcus aureus*.
• GEL/SA-*O.* *dichroanthum* hydrogels potent antifungal effects against Candida albicans.

## Graphical abstract

**Figure:**
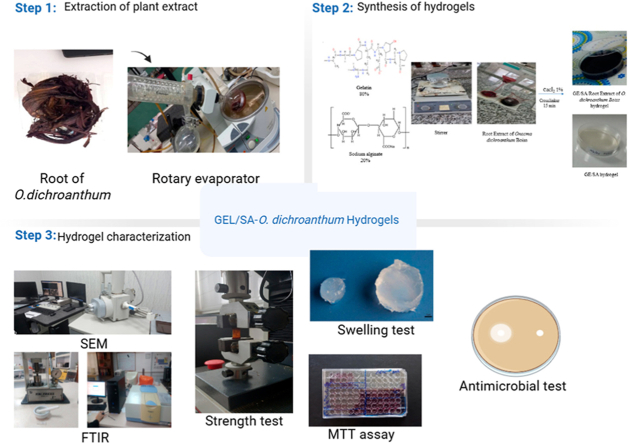

## 1 Introduction

Wound management is a global challenge, costing billions annually. Advanced dressings—gauze, hydrogels, foams, and specialized materials—protect wounds and support healing. Despite progress, current dressings still have limitations [1]. Conventional dressings don't adapt to changing wound conditions, and there's a shortage of dressings for highly mobile areas like joints and the neck [2]. Discomfort during dressing changes is often overlooked. The need for innovative, smart dressings that adapt, comfort patients, and serve diverse needs is growing [3].

Hydrogels are promising wound dressings due to biocompatibility, strong mechanics, and self-healing. While antibiotics are common, resistance and reduced effectiveness from long or repeated use highlight the need for novel, potent antibacterial agents [4]. A promising strategy is stimuli-responsive, intelligent formulations with high encapsulation efficiency for controlled, on-demand antimicrobial release [5]. Antimicrobial hydrogels are emerging as alternatives to conventional antibiotics, inhibiting contamination and sometimes bypassing resistance. They incorporate agents such as natural extracts and metal/metal oxide nanoparticles to boost antimicrobial properties [6]. Natural polysaccharides, in particular, have been widely utilized in the preparation of hydrogels endowed with antimicrobial, antioxidant, self-healing, and adhesion capabilities [7]. Sodium alginate (SA) is a biocompatible, biodegradable polysaccharide from brown algae. It forms highly porous, pH-responsive hydrogels and becomes a negatively charged polyelectrolyte in neutral/alkaline conditions, making it ideal for bio-scaffolds in diverse biomedical applications [8]. Gelatin (Gel), a collagen byproduct, is cytocompatible and supports cell adhesion. Its low cost makes it a viable, economical alternative to collagen, helping reduce costs in cell-cultured meat production [9].

Medicinal and aromatic plants, especially as antioxidants, have gained global prominence [10,11]. Excessive antibiotic use has driven the spread of resistance genes and bacterial resistance [12,13]. Boraginaceae includes about 100 genera and 2000 species across temperate and tropical regions [14]. The wound-healing potential of this family stems from antibacterial, antiviral, antioxidant, and anti-inflammatory activities, mainly due to phenolics like flavonoids, phenolic acids, and naphthoquinones (e.g., alkannin and shikonin) [15]. *Onosma dichroanthum* Boiss. (*Boraginaceae*), known locally in northern Iran as "*Hava Chobeh*," is one of the most significant medicinal plants. Its red root extract has been employed in traditional medicine—either alone or in combination with other medicinal herbs—as an antiseptic and anti-inflammatory agent for treating wounds and burns [16].

A study prepared and characterized gelatin/sodium alginate hydrogels infused with extracts from *Thymus kotschyanus* aimed at wound healing. These hydrogels showed good mechanical properties, elasticity, biocompatibility, hemocompatibility, and antimicrobial activity against common wound pathogens, suggesting promise for wound dressing applications [17].

Several recent studies developed sodium alginate-gelatin hydrogels incorporating plant oils (e.g., *Santalum album* oil) or small molecule compounds for advanced wound healing functions such as antibacterial activity, enhanced fibroblast proliferation, and faster wound closure in animal models. These hydrogels showed promising antimicrobial and biocompatible properties [18,19].

Onosma dichroanthum root extracts, particularly cyclohexane extracts, have been shown in prior work to possess strong wound healing activity in vitro through mechanisms including anti-inflammatory effects and stimulation of fibroblast growth. These bioactivities are attributed to components such as naphthoquinone derivatives in the root extract [20].

Because *Onosma dichroanthum* Boiss is native to Iran, readily available, and used in traditional Iranian medicine for treating wounds and burns, it was included in this study and as antibiotic resistance continues to rise, affordable and widely accessible wound dressings become increasingly important, and This study focused on developing and characterizing a gelatin–sodium alginate hydrogel enriched with *Onosma dichroanthum* Boiss. root extract for potential use in wound dressing applications.

## 2 Materials and Methods

### 2.1 Materials

Sodium alginate (Mv = 1.2 × 10^5^, μ = 280 mPas) and gelatin were purchased from Shanghai Chemical Reagent Co. (Shanghai, China). phosphate buffered saline (PBS) tablet (Aldrich, USA) and ethanol (C_2_H_5_OH, 98 % purity) were supplied by Merck (Germany), while calcium chloride (CAS No. 10043-52-4) was procured from Sigma-Aldrich Co. (USA). All other chemicals were of analytical grade, commercially available, and used as received. The roots of *Onosma dichroanthum* were sourced from the local market and identified by the Department of Botany at Tabriz University (HRU), Tabriz, Iran, under reference number 15415 (EAZH). Additionally, mouse fibroblast cells (L-929) and microbial strains, including *Staphylococcus aureus (S. aureus), Escherichia coli (E. coli), Pseudomonas aeruginosa (P. aeruginosa)*, and *Candida albicans (C. albicans)*, were obtained from the Pasteur Institute of Iran.

### 2.2 Herbal extraction

The root of *O. dichroanthum* was cut into small pieces and ground. A total of 18 g of the material was extracted using 70 % ethanol (500 mL) at 40 °C for 72 h in a Soxhlet extractor. The extract was then concentrated using a rotary evaporator (Heidolph, Germany), air-dried for 4–5 days, and subsequently used in further experiments [21].

### 2.3 Synthesis of hydrogels

2 % weight-volume solution of gelatin and sodium alginate was prepared separately. The two polymers were then combined at a ratio of 80:20 and placed on a magnetic stirrer for 24 h. After this period, a uniform polymer solution was obtained. 2.5 % concentration of *O. dichroanthum* root extract was incorporated into the polymer mixture and stirred at room temperature for 60 min using a magnetic stirrer until fully dissolved. The resulting hydrogel solution was then poured into a Petri dish and allowed to dry under laboratory conditions. Once dried, it was immersed in a 1 % calcium chloride solution for 15 min to facilitate sodium alginate cross-linking.

## 3 Hydrogel characterization

### 3.1 Scanning electron microscopy

The surface morphology of the produced hydrogel was examined using scanning electron microscopy (SEM) (TESCAN-Vega3, Czech Republic). To prepare the samples, the hydrogels were frozen in liquid nitrogen and fractured using tweezers. The cross-sections of the broken samples were then coated with a thin layer of gold for SEM analysis.

### 3.2 Fourier transform infrared spectroscopy

The functional groups and bonds present in the samples were analyzed using FT-IR spectroscopy (Spectrum RX1 FTIR system, PerkinElmer, USA). The samples were finely ground and pressed into thin tablets for examination. FT-IR absorption peaks were recorded within the wavelength range of 400–4000 cm^−1^, and the bond types were identified by analyzing the absorption bands.

### 3.3 Swelling studies

A swelling test was conducted to evaluate the adsorption and swelling capacity of the Gel/SA hydrogel containing *O. dichroanthum* root extract. First, the prototype's initial weight was recorded, and the hydrogel film was immersed in phosphate-buffered saline (PBS) at 37 °C for 5 min. The inflation rate of the sample was measured at time intervals of 30, 60, 90, 120, 150, 180, and 210 min. After reaching equilibrium, the sample was weighed, and the water absorption or swelling rate was determined using the following equation:

$$Swelling\;ratio\;\left(\%\right)=\left(\frac{W_{1}-W_{0}}{W_{0}}\right)\times 100$$

where W_0_ represents the dry weight of the sample and W_1_ is the weight of the sample after water absorption.

### 3.4 Water vapor transmission rate

20 mm diameter section of each *Hydrogel* was placed in separate beakers containing 25 mL of double-distilled water. Additionally, a tube containing 25 mL of double-distilled water without a cap was used as a control. Initially, the test tubes were weighed along with the water and the corresponding samples. The samples were then incubated at 37 °C and 40 % humidity for 24 h. After this incubation period, the samples were reweighed, and the amount of water vapor loss was determined using the following equation.

$$\text{WVTR}=\frac{W_{i}-W_{f}}{A}$$

W_i_ (initial weight of the system; test tube, Water and polymer film (, W_f_ is the final weight of the system after reduction and A is the area of the mouth of the bottle [22]. The WVTR (g/m^2^⋅day).

### 3.5 Biodegradability studies

Degradation tests were performed by immersing hydrogel samples (1 × 1 cm) in 10 mL of PBS (pH 7.4). The hydrogel samples were initially weighed and placed in Falcon tubes containing 10 mL of PBS solution, then stored in an incubator at 37 °C. At predetermined time intervals (1, 3, 5, 7, and 14 days), samples were removed from the PBS, dried in an oven for 2 h, and reweighed. The weight loss was then calculated using the following equation:

$$\text{Degradation}\;\text{ratio}\;\left(\%\right)=\left(\frac{W_{i}-W_{f}}{W_{i}}\right)\times 100$$

where Wi represents the initial weight of the hydrogel and W_f_ denotes the final weight after degradation.

### 3.6 Strength studies

Hydrogel samples were prepared according to ASTM D00882 standards, with dimensions of 1 × 6 cm. The tensile strain rate was set to 50 mm/min, with a jaw separation distance of approximately 3 cm by STM-1 DBBP-100 KOREA. The percentage of elongation and elastic modulus of the hydrogel were determined using the stress-strain diagram and tensile strength measurements [23].

$$TS=\frac{Fmax}{A}$$

### 3.7 Porosity studies

To determine the porosity of hydrogels, the samples were immersed in ethanol until they reached a saturation state. Porosity was then calculated using the following equation:

$$P\;\left(\%\right)=\left(\frac{V_{1}-V_{3}}{V_{2-}V_{3}}\right)\times 100$$

Briefly, a hydrogel sample with weight W was placed in a graduated cylinder containing ethanol. The initial volume of ethanol was recorded as V_1_, while the total volume of ethanol and hydrogel was noted as V_2_. After 1 h, the hydrogel sample was removed, and the remaining ethanol volume was recorded as V_3_.

### 3.8 Hemocompatibility studies

The hemocompatibility of the Gel/SA - *O. dichroanthum* was evaluated using a hemolysis assay. Fresh anticoagulated blood (2.50 mL) from a human volunteer was stabilized with heparin, diluted with 5.00 mL of normal saline, and centrifuged at 9000 rpm for 5 min to isolate red blood cells (RBCs). The RBCs were washed three times with normal saline, then suspended in 20 mL of normal saline. 1 mL RBC suspension was treated with varying hydrogel concentrations (100, 200, 300, 400, 500, and 600 μg/mL). The samples were incubated at 37 °C for 3 h, followed by centrifugation at 9000 rpm for 5 min. The supernatant (100 μL) from each sample was transferred to a 96-well plate, and absorbance was measured at 577 nm using a microplate reader (Biotek, VT, USA) The hemolytic degree was calculated using the following equation:

$$\text{Hemolysis}\;\left(\%\right)=\left(\frac{\text{ODs}-\text{ODnc}}{\text{ODpc}-\text{ODnc}}\right)\times 100$$

where OD_s_ represents the absorbance of the sample, OD_n_c is the absorbance of the negative control (normal saline), and OD_p_c is the absorbance of the positive control (deionized water) [24].

### 3.9 Antibacterial and antifungal activity

The antibacterial activity of the prepared hydrogels was evaluated against *S. aureus* ATCC 25923 (Gram-positive), *E. coli* ATCC 25922, and *P. aeruginosa* ATCC 27853 (Gram-negative). Additionally, an antifungal test was conducted against *C. albicans* ATCC 10231 using the disk diffusion method with Mueller–Hinton Agar (MHA) medium (Merck, Germany). To prepare agar plates, 38 g of MHA medium was dissolved in 1 L of distilled water and sterilized by autoclaving. The sterilized medium was then poured into plates and allowed to solidify. Subsequently, 100 μL of a prepared bacterial suspension (10^8^ CFU/mL) was evenly spread onto each agar plate. Circular hydrogel samples (2 cm in diameter) were placed on the plates and incubated at 37 °C for 24 h. Following incubation, the inhibition zones—regions without bacterial growth surrounding each hydrogel sample—were measured using a Vernier caliper and recorded in millimeters (mm). Gentamicin (20 μg/disk) (Mast.UK) was used as a positive control in the disk diffusion assay.

### 3.10 MTT assay

The MTT assay was conducted using 3-(4,5-Dimethylthiazol-2-yl)-2,5-Diphenyltetrazolium Bromide (MTT reagent), PBS, Fetal Bovine Serum (FBS), and Dulbecco's Modified Eagle Medium/Nutrient Mixture F-12 (DMEM/F-12), all of which were obtained from Sigma-Aldrich (USA). Fibroblast cell lines used in this study were sourced from the Pasteur Institute of Iran (IPI). The fibroblast cells were cultured in DMEM/F-12 medium supplemented with 10 % FBS, 0.25 μg/mL amphotericin, 100 μg/mL streptomycin, and 100 U/mL penicillin. To ensure optimal growth and maintain over 80 % confluence, the culture medium was refreshed every two days. The cells were then trypsinized and seeded at a density of 2 × 10^3^ cells per well in 96-well plates. To assess cytotoxicity, the MTT assay was performed on fibroblast cells exposed to either Blank Hydrogel (Gel/SA) or Hydrogel containing *O. dichroanthum* root extract (Gel/SA/*O. dichroanthum*). The cells were categorized into three experimental groups: **G1:** Control (untreated fibroblast cell line), **G2:** Blank Hydrogel (Gel/SA), **G3:** Gel/SA/Root Extract of *O. dichroanthum*. To eliminate residual media components, the control and experimental groups were washed with sterile PBS. Subsequently, 50 μL of MTT solution (5 mg/mL in PBS) was added to each well and incubated for 4 h at 37 °C under a humidified atmosphere containing 95 % air and 5 % CO_2_. Following incubation, 200 μL of DMSO was introduced to dissolve the formed formazan crystals. The absorbance of each well was measured at 570 nm using a microplate reader (ELx808, Lonza Biotech Co., Switzerland). The cell viability percentage was then calculated using the following equation:

$$Cell\;viability\;\left(\%\right)=\frac{A570\;\left(sample\right)}{A570\left(control\right)}\times 100$$

### 3.11 Statistical analysis

Cytotoxicity analysis was performed using GraphPad Prism v.8.0 software (GraphPad Software Inc., San Diego, CA). Statistical comparisons among groups were conducted using a one-way ANOVA, with P-values ≤0.05 considered statistically significant.

## 4 Result and discussion

### 4.1 Morphology

**Fig. 1:**
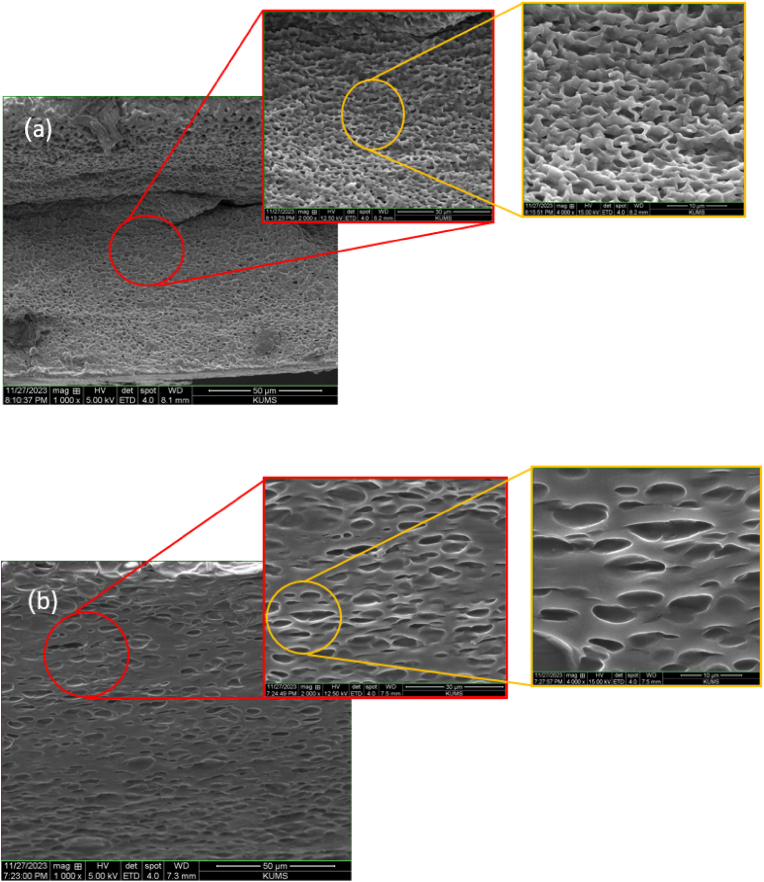
SEM images of the hydrogel: a) GEL/SA, b) GEL/SA/*O. dichroanthum*.

The examination of the structural and compositional characteristics of the gelatin –alginate hydrogels using SEM, (Fig. 1), demonstrated a porous and interconnected framework. SEM imaging revealed that hydrogels containing the extract, The presence of aggregated plant extract particles may disrupt the polymer matrix, potentially influencing durability and degradation rates [25]. The addition of the extract to the hydrogel led to notable alterations in the pore morphology. These modifications are likely due to the interactions between the components of the extract and the hydrogel matrix, which may have influenced the arrangement of the polymer network [26]. Although alterations in pore morphology were noted, the hydrogel preserved its 3D structure. The extract's presence caused a minor swelling or disruption of the polymer network, resulting in an increased average distance between the crosslinks. This formation of a more open structure may enhance the hydrogel's capacity to absorb and release substances, which could be advantageous based on the specific application. This suggests a favorable compatibility between the extract and the hydrogel matrix, facilitating a uniform distribution of the extract throughout the hydrogel [27].

### 4.2 Swelling

**Fig. 2:**
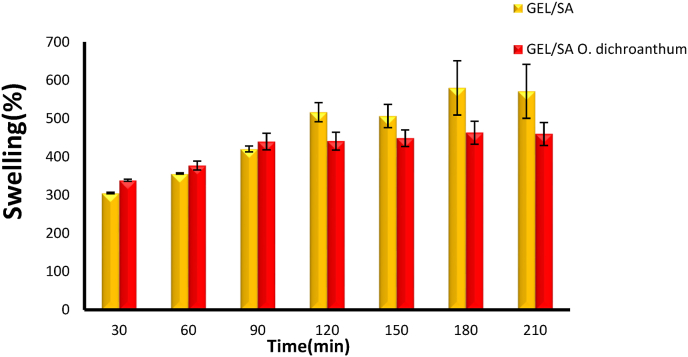
Swelling of hydrogels.

The swelling characteristics of hydrogel films are essential in controlling the release kinetics and effectiveness of the encapsulated extracts. A reduced swelling ratio leads to an extended release process, whereas an increased ratio hastens it. Therefore, the swelling behavior is a crucial factor in customizing hydrogel films for particular applications [28]. The swelling characteristics of the hydrogel were evaluated by measuring its peak water absorption at different time intervals, (Fig. 2). This improved swelling capacity is due to the presence of amino, hydroxyl (-OH), and carboxyl (-COOH) functional groups within the Gel/SA hydrogel structure, which promote significant hydrogen bonding with water molecules [29]. The hydrophilic and hydrophobic characteristics of polymers and incorporated moieties influence the swelling behavior of hydrogels. All hydrogels exhibited peak swelling for a duration of 3 h, after which their swelling diminished, potentially due to polymeric erosion as noted in earlier research [30]. Conversely, the existence of hydrophobic compounds in plant extracts affects the swelling of hydrogels by altering the network structure of the gel. These compounds enhance the density of hydrophobic regions within the hydrogel matrix, thereby diminishing its capacity to absorb water and swell [31].

Some hydrogels with lower swelling are deliberately engineered to achieve stability, reduced adhesion to tissues, or specific drug release profiles, still resulting in effective wound healing outcomes [32]. Moderate swelling hydrogels with incorporated bioactive components have demonstrated both accelerated wound healing and mechanical stability. For example, hydrogels with added sulfated alginate showed increased pore size and swelling ratio facilitating exudate absorption and material exchange, enhancing healing rates despite variations in swelling behavior [33].

### 4.3 Degradation

**Fig. 3:**
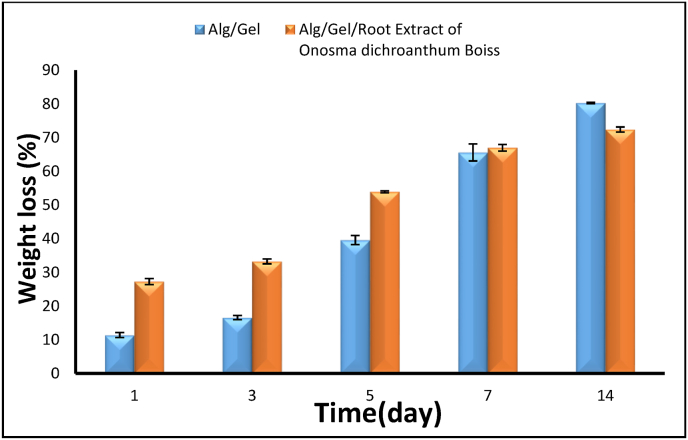
Degradation of hydrogel.

The degradation of hydrogel is a vital aspect of their design, as an ideal hydrogel should gradually decompose in alignment with the regeneration of new tissue. The degradation of the synthesized hydrogels was assessed by tracking their weight loss in PBS at 37 °C over a period of 14 days. (Fig. 3), the rate of biodegradation of the hydrogels increased progressively over time. After 14 days, the GE/SA hydrogel exhibited an 80.26 % degradation rate. In the presence of the extract, the degradation rate was recorded at 72.38 %. Gelatin enhances the degradation process of alginate-based hydrogels, which is further affected by plant-derived compounds. These extracts contain bioactive molecules such as flavonoids and tannins that can interact with the hydrogel matrix, facilitating accelerated biodegradation [34]. Hydrogel-based films are becoming more and more well-known for their capacity to hold onto moisture and provide the ideal conditions for wound healing, which speeds up recovery. Hydrogels based on natural polymers are especially beneficial because they are affordable, biocompatible, and biodegradable [35].

### 4.4 Hemocompatibility

**Fig. 4:**
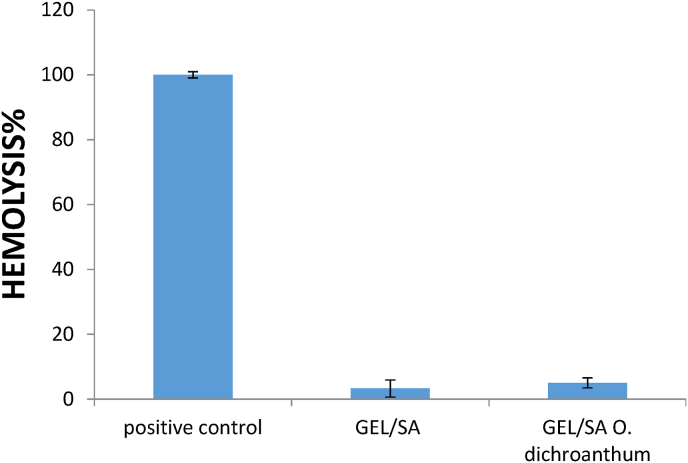
Hemocompatibility studies.

Hemolysis testing is essential for assessing the blood compatibility of biomaterials intended for tissue engineering. Interactions between the hydrogels and blood can disrupt erythrocyte membranes and cause hemoglobin leakage [36]. The two hydrogel formulations were evaluated for hemocompatibility using red blood cells, with results shown in Fig. 4. Both hydrogel variants exhibited negligible hemolysis (<0.5 %), indicating preserved RBC membrane integrity upon contact. PBS served as the negative control and produced no detectable hemolysis, while the positive control produced complete hemolysis (100 %). These findings demonstrate good blood compatibility of the hydrogels and suggest minimal risk of hemolysis in potential clinical applications.

### 4.5 Porosity

**Fig. 5:**
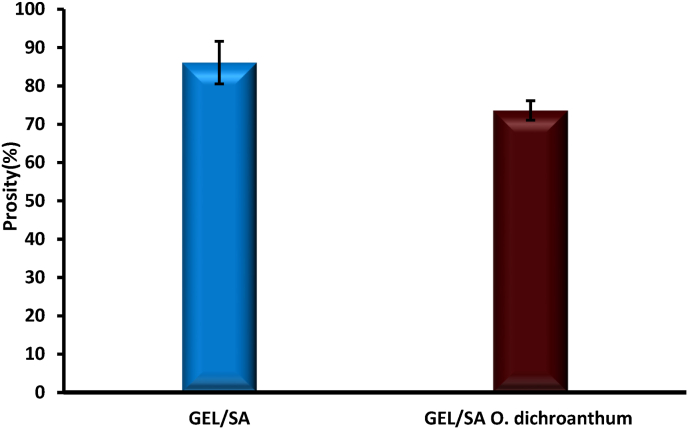
Porosity studies.

Porosity serves as an essential characteristic of hydrogel, affecting cell adhesion, the diffusion of nutrients, mechanical properties, and the integration of tissue [37]. The study of porosity in polymer hydrogel scaffolds demonstrates its significant influence on both mechanical characteristics and biological efficacy. The porosity levels of the hydrogels were assessed using the alcohol displacement technique. Notably, the Gel/SA hydrogel exhibited a high porosity of 86.08 %. However, following the addition of plant extract, a minor reduction in porosity was noted, resulting in a final percentage of 73.56 % (Fig. 5). Alginate, gelatin, and root extract from *O. dichroanthum* Boiss were used to create hydrogel, which was then tested for its potential as a porous scaffold for wound dressing applications. Gelatin and alginate were mixed to improve the mechanical strength, biocompatibility, and biodegradability of the composite. Sodium alginate is known to form robust and stable porous gels through ionic cross-linking with calcium ions, as reported in literature. Despite its bioactive and biocompatible properties that promote cell adhesion, cross-linked alginate gels exhibit limitations such as reduced durability in physiological settings, which impacts their swelling behavior and drug release profiles [[38], [39], [40], [41]].

### 4.6 Strength test

The physicochemical properties of compounds significantly influence their unique characteristics and practical applications, making their evaluation and quantification critical in materials science. Among these properties, mechanical characteristics are particularly vital as they determine how materials respond to external forces [42]. The mechanical properties of Gel/SA hydrogel incorporating the *O. dichroanthum* extract were evaluated via tensile testing Table 1.

**Table 1:** Investigation of the strength of hydrogel.

Hydrogel	Tensile strength (MPa)	Young's modulus (MPa)	Elongation at break (%)
GEL/SA	0.676 ± 0.0045	1.4977 ± 0.0525	46.1335 ± 1.241375
GEL/SA *O. dichroanthum*	0.917 ± 0.0465	1.856 ± 0.0763	49.502 ± 0.52625

Tensile strength up by about 35.7 %, modulus by 23.9 %, elongation by 7.3 %. These indicate that adding the plant extract enhances mechanical properties, making the hydrogel stronger and more flexible. That's important for wound dressings which need to withstand stress without breaking. Higher tensile strength means the hydrogel can handle more stress before tearing. Increased Young's modulus suggests it's stiffer, which might help maintain structure when applied to a wound. Better elongation means it can stretch more before breaking, which is good for movement areas. Results indicated that tensile strength (σ), elongation at break (ε), and Young's modulus (E) decreased upon extract addition. Typically, stronger chemical bonds within the hydrogel network enhance mechanical strength while reducing permeability. The extract's presence improving material elasticity [43]. The specific impact would depend on the concentration and type of extract used, as well as the method of incorporation into the hydrogel. Plant extracts often contain bioactive compounds that can interact with hydrogel matrices. These interactions might alter the crosslinking density or polymer interactions, potentially affecting mechanical properties like strength and stiffness. The extract can modify hydrogel mechanics through several plausible mechanisms, including physical filling that increases crosslink density, additional hydrogen bonding between extract and polymer, ionic or complexation interactions with charged groups, phase separation creating heterogeneous stiff regions, swelling/mesh-size changes that shift elastic and viscoelastic behavior, and possibly covalent or irreversible bonding if reactive groups are present.

### 4.7 Vapor permeability

**Fig. 6:**
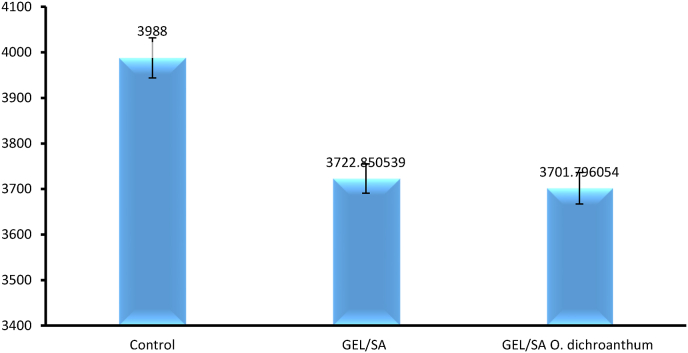
Water vapor transmission rate.

Water Vapor Transmission Rate (WVTR) is a measure of the rate at which water vapor passes through a material per unit area and time. This parameter is used to evaluate the performance of materials in retaining moisture and their permeability to water vapor. Lower WVTR values indicate better resistance of the material to moisture transfer [44]. Alginate and gelatin are widely used in hydrogels due to their biocompatibility and moisture-retention properties. Alginate-based hydrogels often exhibit high WVTR due to their porous structure, which facilitates vapor diffusion [45]. Incorporating bioactive substances like *O. dichroanthum* extract may enhance therapeutic properties but could also modify WVTR by affecting the hydrogel's interaction with water molecules. A notable increase in WVTR was attributed to the enhanced porosity of the Gel/SA hydrogel, (Fig. 6). Conversely, incorporating the extract into the hydrogel led to a slight reduction in WVTR.

### 4.8 MTT assay

**Fig. 7:**
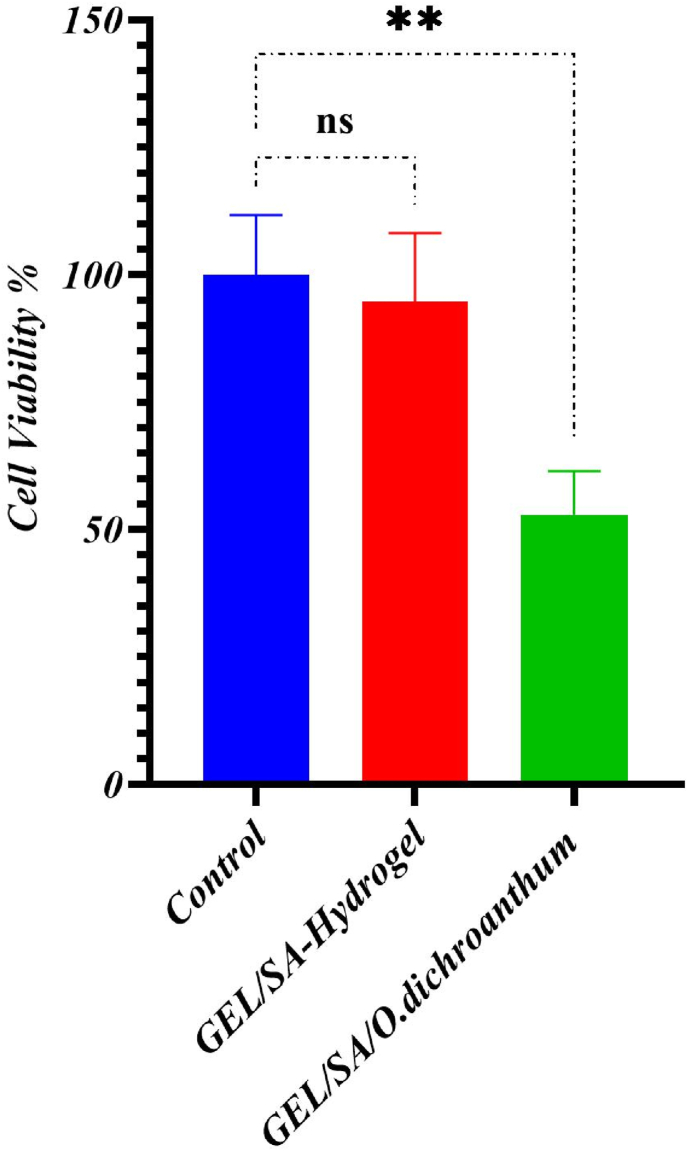
Cell viability.

The biocompatibility and non-toxicity of the manufactured samples are essential prerequisites for their application in the human body. Among the samples, Gel/SA showed superior cell viability. blank hydrogel no toxic but when *O. dichroanthum* (200 μg/mL) added is toxic One of this phenome reason may be is in this study whole extract use and some compounds is toxic for skin cell, and to solve this issue low concentration of herbal extraction needed. (Fig. 7).

### 4.9 FTIR

**Fig. 8:**
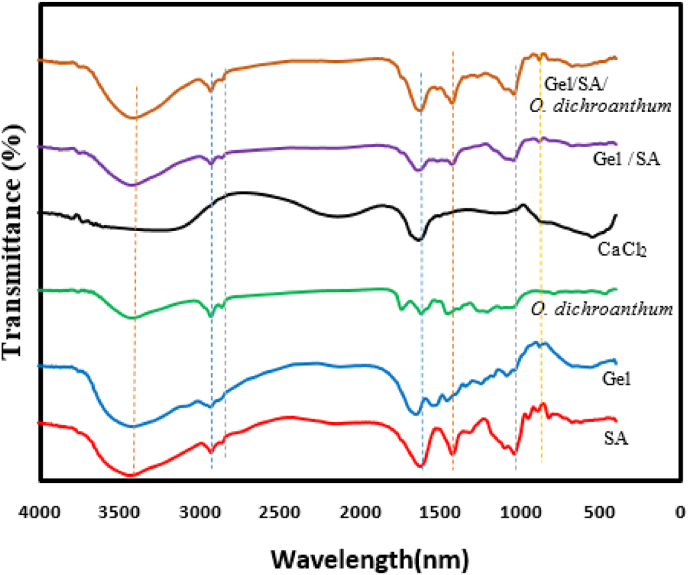
FTIR-spectra.

The FT-IR spectra of the hydrogels were recorded in the range of 500–4000 cm^−1^. Fig. 8 shows the FTIR spectra for alginate, gelatin, *O. dichroanthum*, CaCl_2_, Gel/SA and GE/SA/*O. dichroanthum*. The absorption patterns recorded in the hydrogel containing *O. dichroanthum*. extract was remarkably similar to those in the hydrogel without extract. This observation confirms the absence of significant interactions between the matrix forming the hydrogel and the extract. The FTIR spectrum of Gel/SA/*O. dichroanthum* showed the (OH) stretching absorption band at 13398 cm^−1^. The (C=O) absorption band was shown at 11612 cm^−1^ and (C–H) bond at 2920 cm^−1^, (C–O) band at 1082–1033 cm^−1^, which were similar to the absorption bands in alginate, gelatin, extract but with a slight shift in the position and intensity of the peaks [46,47]. In Gel/SA and Gel/SA/*O. dichroanthum*, the –OH/N–H bands become broader and shift to lower wavenumbers, evidencing strong hydrogen bonding between alginate carboxyl (COO–), gelatin –NH_2_, and polyphenolic –OH groups [48]. Polyphenols contribute extra –OH groups, intensifying and sometimes slightly shifting this band, reflecting their interaction with the biopolymer matrix.

C=O Stretching (Amide I, ∼1645–1620 cm^−1^), Gelatin has a clear Amide I band (∼1645 cm^−1^). In the composite, this peak shifts to lower wavenumbers and may broaden, indicating new hydrogen bonds or ionic interactions (e.g., COO– of SA with NH_3_^+^), and incorporation of polyphenols introduces new C=O features [49]. Stronger or shifted Amide I peaks directly relate to increased crosslinking/coupling, which means reduced mobility. Carboxylate Stretching (COO–, 1400–1600 cm^−1^): Addition of CaCl_2_ (or crosslinking ions) often causes splitting or shifting in symmetric/asymmetric COO– stretches, confirming ionic crosslinking [34]. Increased hydrogen bonding and crosslinking (evidenced by broader and shifted –OH/NH, C=O peaks) restricts polymer chain movement, resulting in higher mechanical strength (Young's modulus) but lower swelling ratio [50]. Gel/SA/*O. dichroanthum*, with pronounced peak shifts and broadenings, likely have extensive network formation, translating to firmer gels with reduced swelling—ideal for wound dressing or drug delivery context. Lower crosslinking (less peak shifting) is associated with softer hydrogels and higher swelling. For overlapping regions (particularly 3200–3500 cm^−1^ and 1600–1700 cm^−1^), deconvolution or second-derivative FTIR can clarify individual –OH, –NH, and C=O contributions and more precisely track shifts due to hydrogen bonding or crosslinking events [49]. Such analyses offer quantitative insight into the balance between physical and chemical crosslinks, vital for fine-tuning mechanical and swelling properties. FTIR analysis of alginate hydrogels confirmed the physical incorporation of the extract without chemical bonding, ensuring the preservation of the polymer's intrinsic properties. Hydrophobic components present in extracts can influence cross-link density and polymer interactions, potentially reducing elasticity, increasing brittleness, and diminishing the swelling capacity of loaded alginate hydrogels. Additionally, incorporating *O. dichroanthum* extract into the hydrogel enhances its antibacterial properties, effectively preventing bacterial infections in wounds [51].

### 4.10 Antimicrobial activity

The hydrogel root extricate of *O. dichroanthum* Boiss appears direct antimicrobial action against *S. aureus* (9 mm zone of hindrance) but no activity against *P. aeruginosa* or *E. coli*. (Table 2).

**Table 2:** Inhibition zones detected for hydrogel.

Organisms	GM (20 μg/disk) (mm)	GEL/SA Control (mm)	GEL/SA *O*. *dichroanthum* (mm)
*S. aureus*	18	0	9
*P. aeruginosa*	17	0	0
*E. coli*	20	0	0

**Fig. 9:**
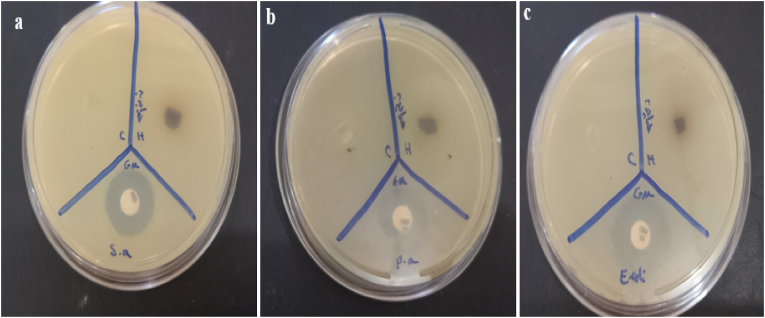
Antibacterial activity of hydrogel **A:***Staphylococcus aureus***B***Pseudomonas aeruginosa* and **C:***E*. *coli*, Gentamicin (20 μg/disk) disks as positive controls.

The root extract appeared a 9 mm zone of hindrance, showing direct antibacterial movement against *S. aureus*. This proposes that the extricate contains bioactive compounds that can hinder the development of Gram-positive microscopic organisms. no antibacterial impact against these Gram-negative microscopic organisms. This can be due to the nearness of an external layer in Gram-negative microbes, which frequently makes them more safe to plant-derived compounds. Gram-positive microbes (like *S. aureus*) have a less complex cell divider structure, making them more vulnerable to certain plant extricates. Gram-negative organisms (like P. aeruginosa and E. coli) have an additional external layer (outer membrane) and active efflux pumps, that can act as an obstruction to numerous antimicrobial compounds. The root extricate of *O. dichroanthum* Boiss may contain compounds (e.g., phenolic, flavonoids, or alkaloids) that are compelling against Gram-positive microbes but not against Gram-negative microscopic organisms. The root extract's action is altogether weaker compared to gentamicin, but it still demonstrates potential as a characteristic antibacterial operator, especially against Gram-positive microbes. The root extricate of *O. dichroanthum* Boiss illustrates specific antibacterial movement (Fig. 9), especially against Gram-positive microscopic organisms like *S. aureus*. Whereas its action isn't as solid as customary anti-microbial like gentamicin, it holds guarantee as a common antimicrobial specialist. Advance investigate is required to completely get it its potential and optimize its utilize. traditional method of wound care Biomaterials act as transient barriers to stop infections and manage bleeding. Improving their qualities can greatly speed up the healing process. The ideal dressing for a wound should keep the area wet, absorb extra fluid, and protect the wound from microbiological contamination [52].

### 4.11 Antifungal activity

**Fig. 10:**
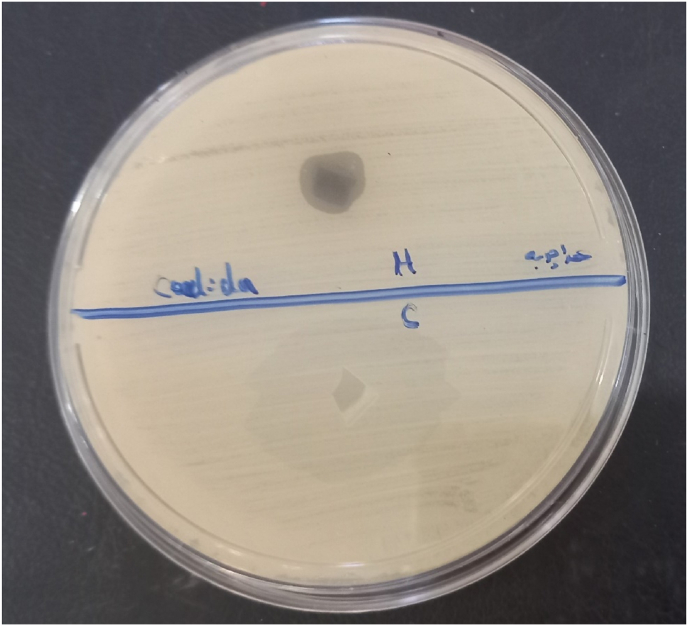
Antifungal activity root extract of *O. dichroanthum* Boiss (10 mm).

In this study GEL/SA *O. dichroanthum* have notable antifungal activity(10 mm) against *c. albicans* The root extricate may contain compounds like phenolic, flavonoids, terpenoids, or alkaloids, which are frequently related with antifungal properties. Whereas the antifungal action of the root extricate of *O. dichroanthum* Boiss isn't however affirmed, it is worth exploring given the potential of plant extricates to show broad-spectrum antimicrobial properties (Fig. 10). Given the increasing resistance of fungal pathogens like *C. albicans* to conventional antifungal agents, exploring plant-derived compounds offers a promising alternative. The observed antifungal effect in this study warrants further investigation, including phytochemical profiling and mechanistic studies, to identify and isolate the active constituents responsible for the antifungal activity [53]. Comparative research indicates that extracts from various *Onosma* species exhibit significant antimicrobial properties attributable to compounds such as naphthoquinones (e.g., alkannin and shikonin derivatives), phenolics, and flavonoids. For example, methanol extracts of Onosma roots have demonstrated notable antifungal activity, sometimes with inhibition zones comparable to or exceeding standard antifungals against Candida species. One study showed that the methanolic root extract methanol exhibited a higher inhibition diameter against Candida glabrata than the antibiotic Nystatin, underlining the potency of such extracts. The presence of alkannin/shikonin compounds is particularly linked to antimicrobial and anti-inflammatory effects that support antifungal activity [54,55].

## 5 Conclusions

We fabricated a 3D Gel/SA/*O. dichroanthum* hydrogel as a wound dressing. Characterization showed a porous, interconnected structure with uniform dispersion of the extract throughout the hydrogel. *In vitro* assays demonstrated good biocompatibility. Incorporation of the extract conferred potent antibacterial activity to the hydrogel. Collectively, the material exhibited favorable physicochemical and biological properties and shows promise as an ideal wound dressing.

## CRediT authorship contribution statement

**Hadi Hossainpour:** Writing – review & editing, Writing – original draft, Visualization, Methodology, Investigation, Formal analysis, Data curation. **Soheila Zare:** Writing – review & editing. **Hosna Alvandi:** Methodology. **Ramin Abiri:** Writing – review & editing. **Faranak Aghaz:** Methodology, Investigation. **Elham Arkan:** Project administration. **Amirhooshang Alvandi:** Writing – review & editing, Writing – original draft, Project administration, Investigation.

## Ethical consideration

This study was ethically approved by the Kermanshah University of Medical Sciences, Institutional Review Board (IR.KUMS.AEC.1401.014)

## Funding statement

This research was funded by a grant (NO. 4010198) from the Student Research Center, 10.13039/501100022299School of Medicine, 10.13039/501100005317Kermanshah University of Medical Sciences, Kermanshah, Iran.

## Declaration of competing interest

The authors declare that they have no known competing financial interests or personal relationships that could have appeared to influence the work reported in this paper.

## Data Availability

The data that has been used is confidential.
